# Meta-ethnography in healthcare research: a guide to using a meta-ethnographic approach for literature synthesis

**DOI:** 10.1186/s12913-020-06049-w

**Published:** 2021-01-08

**Authors:** Raabia Sattar, Rebecca Lawton, Maria Panagioti, Judith Johnson

**Affiliations:** 1 University of Leeds, Leeds, LS2 9JT UK; 2 Bradford Institute for Health Research, Bradford, BD9 6RJ UK; 3 National Institute of Health Research for Primary Care Research, Manchester Academic Health Science Centre, University of Manchester, Manchester, M13 9PL UK

**Keywords:** Meta-ethnography, Research methods, Qualitative synthesis, Qualitative health research

## Abstract

**Background:**

Qualitative synthesis approaches are increasingly used in healthcare research. One of the most commonly utilised approaches is meta-ethnography. This is a systematic approach which synthesises data from multiple studies to enable new insights into patients’ and healthcare professionals’ experiences and perspectives. Meta-ethnographies can provide important theoretical and conceptual contributions and generate evidence for healthcare practice and policy. However, there is currently a lack of clarity and guidance surrounding the data synthesis stages and process.

**Method:**

This paper aimed to outline a step-by-step method for conducting a meta-ethnography with illustrative examples.

**Results:**

A practical step-by-step guide for conducting meta-ethnography based on the original seven steps as developed by Noblit & Hare (Meta-ethnography: Synthesizing qualitative studies.,1998) is presented. The stages include getting started, deciding what is relevant to the initial interest, reading the studies, determining how the studies are related, translating the studies into one another, synthesising the translations and expressing the synthesis.

We have incorporated adaptations and developments from recent publications. Annotations based on a previous meta-ethnography are provided. These are particularly detailed for stages 4–6, as these are often described as being the most challenging to conduct, but with the most limited amount of guidance available.

**Conclusion:**

Meta-ethnographic synthesis is an important and increasingly used tool in healthcare research, which can be used to inform policy and practice. The guide presented clarifies how the stages and processes involved in conducting a meta-synthesis can be operationalised.

## Background

The range of different methods for synthesising qualitative research has grown in recent years [1]. There are now a number of different qualitative synthesis methods including qualitative meta-synthesis, narrative synthesis, thematic synthesis, interpretative synthesis, grounded theory and meta-ethnography. A qualitative synthesis is defined as *‘any methodology whereby study findings are systematically interpreted through a series of expert judgements to represent the meaning of the collected work’* [2]*.. In a qualitative synthesis the findings of qualitative studies are pooled* [2]*. The use of some types of qualitative syntheses allow for the inclusion of mixed-methods and quantitative research studies alongside qualitative studies.* A qualitative synthesis approach can be used to examine the available literature, and review and integrate the primary research studies related to a specific question or phenomenon, to reveal deeper insights or explanations that would not be possible from a single study [3]. The various qualitative synthesis approaches mentioned above differ in their purposes, philosophical traditions and whether they primarily aggregate or re-interpret the study findings [4, 5]. Meta-ethnography is an inductive, interpretative approach upon which most interpretative qualitative synthesis methods are based [6] and is the most commonly utilised qualitative synthesis approach in healthcare research [7].

Meta-ethnography is particularly suited to developing conceptual models and theories [8]. This method of qualitative synthesis is often chosen over alternative approaches as it is more suitable for the development of analytical rather than descriptive findings [9]. A meta-ethnography differs from other qualitative synthesis approaches as the reviewer re-interprets the conceptual data (themes, concepts or metaphors) created by the authors of the primary study whilst taking into account the primary data (participant quotes) using a unique translation synthesis method in order to transcend the findings of individual study accounts and create higher order themes [10, 11]. Meta-ethnographic reviews offer greater description of methods and higher order interpretation compared to conventional narrative literature reviews [12]. In health sciences, meta-ethnographies can be used to generate evidence for healthcare and policy [13]. A meta-ethnographic synthesis approach is suitable when researchers are interested in conceptual or theoretical understandings of a particular phenomenon. Unlike some qualitative synthesis approaches which allow the inclusion of mixed-methods design studies (such as thematic synthesis and interpretative synthesis), a meta-ethnographic approach enables only the inclusion of qualitative studies. A meta-ethnography can include multiple study designs, whereas other approaches such as grounded theory require only the inclusion of similar study approaches [14].

Although meta-ethnography is a widely used qualitative literature synthesis method within healthcare research, it is poorly demarcated and there is a lack of clarity surrounding the description of the data analysis process. A number of reviews have used this approach [15–20] but do not provide a fully rigorous description of the stages involved in the analysis process. Given the value of qualitative meta-synthesis in integrating the findings from multiple studies into a higher conceptual level, it is important to provide detailed guidance on each of the steps involved in conducting a meta-ethnography. This paper aims to fill this gap by outlining a step-by-step method for conducting meta-ethnography. We describe our interpretation of each of the seven steps outlined by Noblit & Hare [10] who first proposed this approach. We also incorporate adaptations and developments by recent researchers [21] and provide annotations where applicable to assist in describing the stages involved.

The worked example we are using is a published meta-ethnography (reference and author names omitted for author anonymity during peer review). Where applicable, illustrative examples from this review are provided alongside the each phase to demonstrate the process.

Within a meta-ethnographic synthesis, the process of translation is key and unique. It is defined as ‘comparing the metaphors and concepts in one account with the metaphors and concepts in others’ [10]. A meta-ethnography should involve a reciprocal and refutational translation, where possible combined with a line of argument synthesis [22, 23]. Reciprocal translation occurs when concepts in one study can incorporate those of another [22], whereas a refutational translation explains and explores differences, exceptions, incongruities and inconsistencies [1, 22, 24]. Reviewers often overlook refutational translation [24, 25]; however studies may refute each other [26, 27] or concepts within studies may refute one another [27, 28]. Therefore it may be possible to conduct both a reciprocal and refutational translation [22]. A line of argument synthesis is not an alternative to conducting a translation but is viewed as the next stage of analysis [23]. A line of argument synthesis is the translation of accounts that interpret different aspects of the same phenomenon under study, which results in producing a whole that is greater than the sum of its individual parts [10, 15]. Although Noblit & Hare [10] describe meta-ethnography as a seven step process, it is important to acknowledge that this process is iterative and the phases are not discrete but may overlap and run in parallel [10]. A meta-ethnography reporting tool, eMERGE has very recently been developed, and provides a framework for reviewers to follow when reporting the important aspects of a meta-ethnography [22].

## Methods

In order to identify relevant literature to inform the present guide, we searched for articles which described an evaluation or discussed methodological issues in conducting a meta-ethnography or provided guidance for reporting a meta-ethnography. We then scanned the reference lists of relevant articles to identify further relevant literature. We also drew on the results from two recent systematic reviews [23, 29]. As such, while the searches conducted for the present article were not systematic, the guide reflects recent methodological recommendations in the wider methodological literature. All relevant articles were read and recommendations were noted; where any disagreement between authors of papers was apparent, guidance which was based on systematic reviews of the evidence rather than individual reflections was prioritised.

## Results

### Doing a meta-ethnographic synthesis: a step-by-step guide with illustrated examples

#### Phase 1: getting started

The initial stage requires the authors to identify an area of interest [10]. The reviewers need to consider if a synthesis of the topic is required and whether a qualitative synthesis and the meta-ethnographic approach fits with the research question [30]. E.g. A meta-ethnographic synthesis approach is suitable when researchers are interested in conceptual or theoretical understandings of a particular phenomenon. It is also important to determine whether there is a large and growing body of qualitative research in this area, and whether synthesizing qualitative findings can contribute valuable knowledge to the existing literature [31]. As proposed by Campbell and colleagues [32], we emphasize, that at this stage, it is important to establish a team of researchers who have different approaches, opinions and the key skills to conduct the meta-ethnography, as this will add rigour to the meta-ethnographic review.

*Example*

We were interested in the disclosure of adverse events within healthcare; specifically in the perceptions and experiences of patients and healthcare professionals relating to these events. We were aware of the large and growing body of qualitative research in this area. Our searches revealed that there was no qualitative synthesis specific to the experiences of adverse event disclosure. We believed that synthesizing the views, attitudes and experiences of both groups (patients and healthcare professionals) would enable us to understand what patients require from the disclosure conversation and what healthcare professionals currently offer. Our motivation for synthesizing the body of qualitative evidence was to inform future disclosure interventions which were acceptable to patients and practical for healthcare professionals to deliver. Synthesizing qualitative findings can make valuable knowledge accessible to healthcare professionals and policy makers [31].

#### Phase 2: deciding what is relevant to the initial interest

Once you have chosen your topic of interest, phase 2 involves the following steps: a) defining the focus of the synthesis, b) selecting studies for inclusion in the synthesis and locating relevant studies, c) developing inclusion and exclusion criteria and d) quality assessment of the included studies [12].

##### 2a. Defining the focus of the synthesis

An important decision involves deciding whether to include all the studies within your chosen area of interest. It is necessary to find a balance between a review which has a broad scope, and a focus which will yield a manageable number of studies. The scope of a meta-ethnography is more restricted compared to other qualitative narrative reviews. This is due to the avoidance of making gross generalisations across disparate fields [10, 26]. There is currently no agreement to how many studies should be included in the synthesis. Some researchers argue that synthesizing a large number of studies may interfere with the ability to produce a useful interpretative output and could result in an aggregative synthesis [23]. Synthesizing too few studies can result in underdeveloped theories/concepts [24, 28]. A large number of studies have varied from 40 [32] to over 100 [24]. The volume of data, rather than just the number of studies is important and team size and resources will affect the ability to manage this data [22]. It is recognised that focusing on a particular aspect of your chosen topic interest and excluding certain aspects may result in some papers being overlooked. However it is important to make this choice to ensure that you have manageable number of studies [12].

*Example*

Our systematic review question focused on *‘The views and experiences of patients and healthcare professionals on the disclosure of adverse events’.* We focused on studies which examined the views and experiences of patients (and/or family members, members of the general public) and healthcare professionals. We found that qualitative research in the area of adverse event disclosure was limited. As this was an under-researched area, we were able to include all the available qualitative studies in this research area (enabling us to include both patients’ and healthcare professionals’ views on adverse event disclosure).

##### Phase 2b: locating relevant studies

The second important step involves locating potentially relevant qualitative studies by conducting a systematic search of the literature. In order to conduct a systematic search, a well-constructed and comprehensive search strategy needs to be developed. Qualitative searches can yield a large number of search results, which can be daunting and time consuming to screen. One of the ways to make your search strategy more specific is through the use of qualitative search filters. Empirically tested search filters for qualitative studies have been developed [33–35]. However it is possible that some of the potentially relevant studies may be missed when using such filters. Decisions regarding your search strategy and screening depend on your aims and resources available. We advise the use of a librarian for reaching decisions on the content of searches. Multiple databases are utilised to locate relevant articles and this can be further supplemented by hand searching. This is important as it can locate relevant articles which are not indexed or inaccurately indexed, and minimises the risk of missing relevant studies [24].

Some argue that a more purposive sampling approach may be more appropriate [36, 37], which aims to provide a holistic interpretation of a phenomenon, where the extent of searching is driven by the need to reach theoretical saturation rather than to identify all eligible studies [24, 38]. Detailed information on purposive sampling technique is available [24, 28]. Also, to avoid the potential problem of having too few descriptively or conceptually-rich studies, knowledge-building and theory-generating systematic reviewers can conduct expansive searches of the literature [28]. We do not describe here how to conduct a systematic search of the literature, however there are a number of papers which describe this process [39–41].

*Example*

We searched five electronic databases, and our search strategy included a combination of the three major concepts (disclosure, safety incident and experience). We also supplemented the database searches by hand searching relevant journals and reference lists. We chose not to apply qualitative filters in order to capture all the possible relevant articles.

##### Phase 2c: decisions to include studies

A number of factors should be considered when deciding whether to include or exclude studies from the synthesis. An important consideration is the expertise of the review authors and the time available to complete the review [36]. Reviewers should consider the likelihood of excluding valuable insights on the basis of quality, and the contribution of these studies to the development and interpretation of findings. Would excluding such studies affect the coherence of qualitative synthesized findings? [36]. Also, an important consideration is the nature of the primary data which is available to synthesise [23]. Including predominantly thin descriptive data can be problematic as it is difficult to further interpret data which lacks depth [23]. Conceptually rich data which is explanatory, or rich descriptive data which provides sufficient detail to be further developed is suitable for meta-ethnography. Therefore selecting studies based on this suitability is one of the approaches reviewers should consider. Further discussion on decisions to include studies is available [36].

##### Phase 2d: quality appraisal

There is a lack of agreement surrounding the use of quality appraisal for qualitative studies [30]. Some researchers argue there are difficulties with quality appraisal as some aspects of qualitative research are difficult to appraise and therefore depend on subjective judgement [5]. Although this debate continues, we argue that at least some quality appraisal of studies needs to be considered to give an indication of the credibility of the included studies. Critically appraising the studies and assigning numerical scores to indicate level of quality is also useful as it can be used as a way to order the studies for analysis. Previous published qualitative reviews have either used the highest scoring paper as the ‘index study’ [15] or have arranged all the papers in chronological order by date, and used the most recently published paper as the ‘index study’ [42]. One of the limitations is of assigning numerical scores using CASP and the use the highest scoring as an index study is that it focuses on the methodological rather than conceptual strength. Other reviewers have chosen a ‘conceptually rich’ index account [43, 44] however it is unclear how this ‘conceptually rich’ index account should be selected. The different ways of ordering study accounts has yet to be formally empirically compared and there is no guidance for reviewers [23]. However the order could affect the synthesis output [11, 12, 24, 45]. There are different perspectives to the use of tools in the quality assessment of qualitative research [46]. Some recommend the exclusion of studies based on a low-quality assessment and others refute this view and suggest that such tools may not truly assess the meaningfulness and potential impact of qualitative findings [47]. However, we believe that these checklists can equip novice qualitative researchers with the resources to evaluate qualitative research efficiently.

Two common and widely used quality assessment tools are the Critical Appraisal Skills Programme (CASP) and the Qualitative Assessment and Review Instrument (JBI-QARI). The Critical Appraisal Skills Checklist (CASP) provides detailed instructions and decision rules on how to interpret the criteria [48]. This checklist contains a number of questions which help the reviewer to assess the rigour, credibility and relevance of each study [49–52]. All studies are critically appraised and each study is assigned a numerical score out of ten, where a higher score is correlated to a higher quality [15]. The two studies ranked with the highest scores are used as index studies, and can be used as the first studies from which concepts are translated into other studies and therefore shaping the analysis [12]. Similarly, the Qualitative Assessment and Review Instrument (JBI-QARI) is a 10 item checklist which assesses the methodological quality of a study, and determines the extent to which a study has addressed the possibility of bias in its design, conduct and analysis [53]. Some researchers provide guidelines for determining and excluding studies which have major methodological flaws [54]. However, it can be argued that excluding studies based on quality criteria may result in the exclusion of insightful studies. GRADE-CERQual is a recently developed approach which provides guidance for assessing how much confidence to place in findings from systematic reviews of qualitative research [55]. The application of GRADE-CERQual can be helpful for appraising the overall quality of the qualitative synthesis [55] but a quality appraisal of primary studies is required before applying the CERQual tool.

*Example*

We used the CASP checklist to assess the quality of included studies. We chose to use the CASP as it propagates a systematic process through which the strengths and weaknesses of a research study can be identified [56]. The CASP guidelines are easy to follow, especially for novice researchers [56]. We made a decision in advance not to exclude studies with low quality scores. We believed that although some authors may have failed to describe the methods in sufficient detail for us to determine that quality criteria had been met, lack of reporting did not necessarily mean it was poorly conducted research [12]. We did however use the quality rating of the studies in our synthesis approach. The study ranked with the highest score was used as the ‘index study’ and was the first study from which concepts were translated into other studies and therefore shaping the analysis [12].

#### Phase 3: Reading the studies

It is during this phase where the synthesis process begins. First, this involves repeatedly reading the included studies and familiarising yourself with the key concepts and metaphors. It is important at this stage to become as familiar as possible with the content and detail of the included studies. A concept is defined as ‘having some analytical or conceptual power, unlike more descriptive themes [26]. It is important to acknowledge that reading the studies is not a discrete phase; reading occurs throughout the synthesis process. The notion of first, second order and third order constructs [26] are useful in distinguishing the ‘data’ of the meta-ethnography which are defined in Table 1 below.

**Table 1:** Key terms in a meta-ethnography

Primary authors	Refers to the authors of the original primary qualitative studies
Reviewers/team members	Refers to the individuals conducting the meta-ethnography
First order constructs	Represent the primary data reported in each paper (participant quotations).
Second order constructs	The primary authors’ interpretations of the primary data (metaphorical themes or concepts).
Third order constructs	The reviewers higher order interpretations developed from a tertiary analysis of the first and second order constructs.

Once you have read through the chosen studies, the next step involves extracting the ‘raw data’ from the studies for the synthesis. The raw data for a meta-ethnographic synthesis are the first and second order constructs [29, 31]. The data needs to be extracted from each of the studies, which can be done by using a standardised data extraction form [11]. Alternative ways to extract data include creating a list of metaphors and themes [32] or coding concepts in Nvivo; a software programme for the analysis of qualitative data [31]. The data should be extracted verbatim, so there is no risk of losing important data [12] and to preserve the original terminology used by the primary authors. However, some authors of a previous meta-ethnography chose to record summaries of primary author interpretations due to the large number of studies included in their synthesis [12]. However, one of the drawbacks of recording such summaries is that there is the risk of potentially losing important detail.

It is essential at this stage to extract information on study characteristics for each study, using a separate data extraction form as it provides context for interpretations and explanation of each study [57]. This includes information on study sample, data collection methods, data analysis methods, study outcomes and study conclusions.

*Example*

**Fig. 1:**
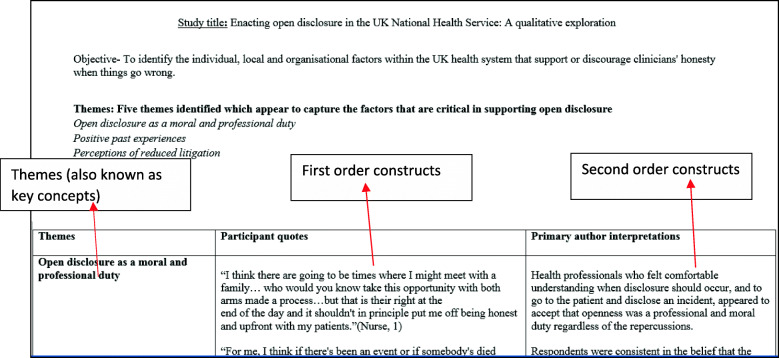
Example of a data extraction table

We have provided an example of a data extraction table we used to extract the raw data (Fig. 1).

#### Phase 4: determining how the studies are related

During this stage, the relationships between the key concepts from the different papers need to be considered. A concept is described as a ‘*meaningful idea that develops by comparing particular instances’* [29, 45]. It is also important that concepts explain and do not only describe the data [29, 45] as one of the aims of qualitative analysis is to develop concepts which help to understand an experience and not just describe it [58]. In order to consider the relationship between concepts from the different studies, you are required to look across the studies for common and recurring concepts. This can be done by creating a list of the themes [10]. These are then juxtaposed against each other to examine the relationships between the key concepts and metaphors these themes reflect and to identify common and recurring concepts. From this list, the themes from the different studies are then clustered into relevant categories, where we grouped common concepts from studies according to the common underlying metaphors, an approach which has previously been used [12, 31, 59]. During this phase it is essential to examine the contextual data about each study. This includes settings, aims and focuses. These newly formed categories are labelled using terminology which encompasses all the relevant concepts they contain. This phase is likely to be iterative, and clusters may be revised through discussions within the review team of how they are related and by making reference to the original text.

Other authors have used diagrams [11, 32] or coding using qualitative analysis software [31]. The use of lists or tables in phase 4 is useful when synthesising a small number of studies, however such an approach would be unwieldy when there are hundreds of concepts, whereas coding in NVivo is efficient [23]. However, the recording of links between concepts within primary studies may be difficult when using NVivo [23].

*Example*

**Fig. 2:**
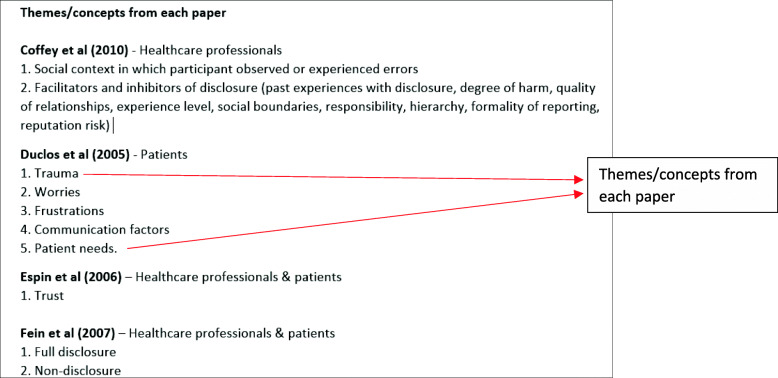
List of key metaphors/concepts from each study

During this phase, for our review we created a list of the themes from each paper (Fig. 2) listed under each study name. As we had included both healthcare professional and patient studies, we also labelled whether the study had included patients, healthcare professionals or both groups.

**Fig. 3:**
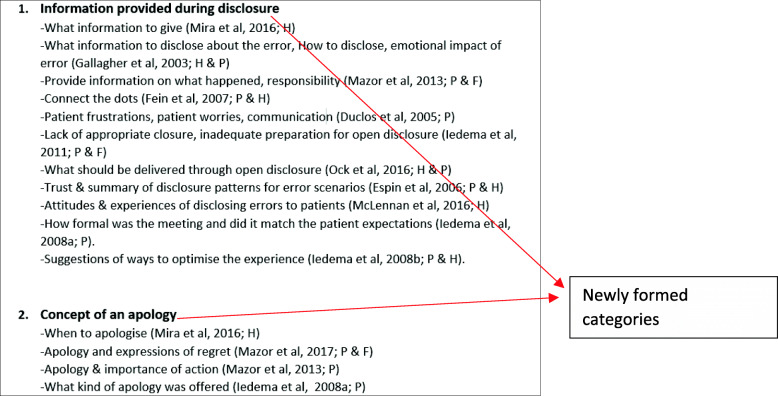
Reducing themes into relevant categories

The next step involved reducing the themes from the different studies into relevant categories (Fig. 3).

It is important to note that the category labels you create during this phase are not the higher third order constructs, but are descriptive labels. The third order constructs are developed within the next two phases. However, the data within each category forms the basis of reciprocal translation or refutational synthesis in the next stages. This approach can work well when you have a manageable number of studies, however this can prove to be challenging when you have a larger number of studies. In previous meta-ethnographies where a large number of studies have been included, a thematic analysis of themes was carried out instead [12].

#### Phase 5: translating the studies into one another

The original method of meta-ethnography suggests that this phase involves ‘comparing the metaphors and concepts in one account with the metaphors and concepts in others’ [10]. However, despite a number of meta-ethnographies being conducted, it is unclear how this should be done and how this phase of the analysis should be recorded. To address this lack of clarity, we will now outline below one way in which this can be done. During this phase, each concept from each paper is compared with all the other papers to check for the presence or absence of commonality. Doing this highlights the similarities and differences between the concepts and metaphors and allows the researcher to organise them into further conceptual categories, which results in the development of the higher third order constructs.

**Fig. 4:**
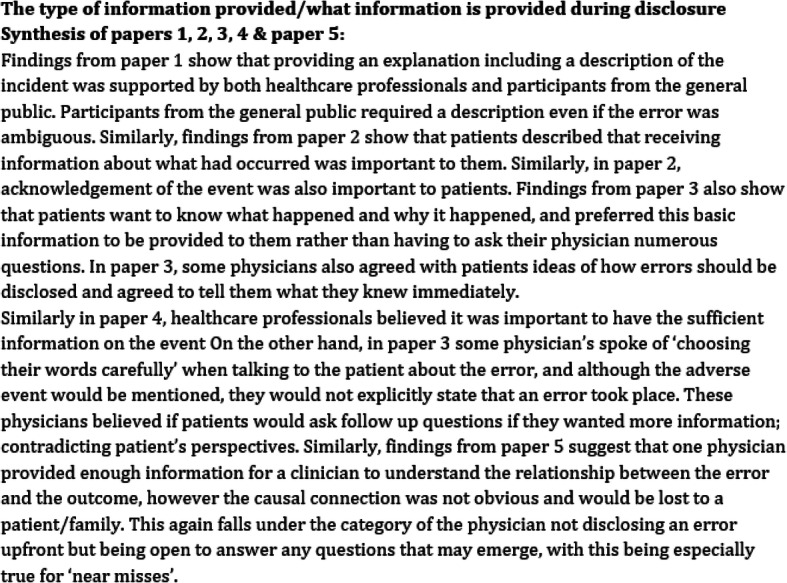
Primary data synthesis of the primary author interpretations

This phase is approached by arranging the studies either chronologically [32] from the highest scoring paper to the lowest scoring paper (where the scores are generated during the quality appraisal process [15]. Arranging the studies chronologically is advised when you are including a large number of papers over a large time span [12, 29]. The order in which studies are compared may influence the synthesis, as earlier papers will have a strong influence on the subsequent development of ideas [60]. The reviewer first starts by summarising the themes and concepts from paper 1. Summarising involves comparing and contrasting the concepts taking into account study contexts. They then summarise the themes and concepts from paper 2, commenting first on what is similar with paper 1 and then what paper 2 may add to paper 1 or where its findings diverge from paper 1 [12, 29]. Next, paper 3 is summarised, considering what is similar to papers 1 and 2, and then noting any areas of divergence and anything that paper 3 adds to the knowledge offered in papers 1 and 2. This process continues until you have synthesised all the papers and produces a synthesis of the primary author interpretations (see Fig. 4) which are useful in aiding with the development of the third order constructs in the next stage.

**Fig. 5:**
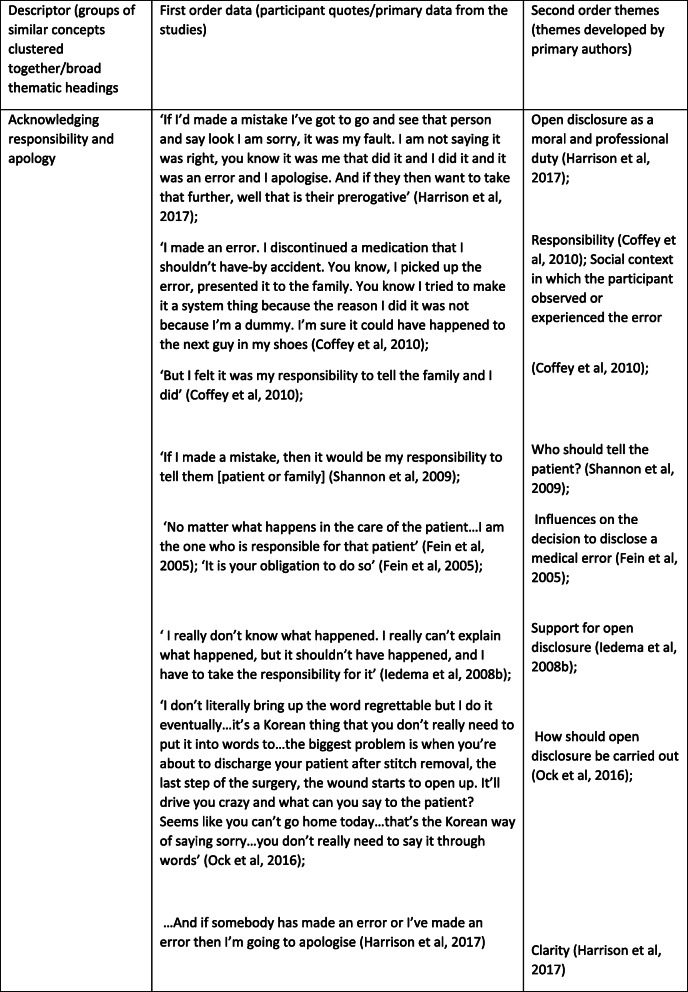
Example of a translations table

Examining the key concepts within and across the studies is similar to the method of constant comparison [29]. During this phase, it is important to refer back to the table of study characteristics you recorded earlier, (country, sample, recruitment method, gender, publication date etc.) to use as a context for the comparisons [15] as well the full papers. This process can also be supported by creating a translations table, as this is a useful way to display this level of synthesis [61] (see Fig. 5 for an example of a translations table). Maintaining a personal journal during this phase of the analysis can help to ensure that the researcher is aware of their position from a theoretical point of view [62]. Discussing the key concepts and their meanings with team members can result in collaborative interpretations.

*Example*

We conducted two separate syntheses; one for the views of patients and one for healthcare professionals, and conducted a line of argument synthesis of all the included studies, therefore we found it useful to have two separate translation tables; one for each group. Part of the translation table for healthcare professionals is shown in Fig. 5 (see example of table below).

#### Phase 6: Synthesising the translations

This phase is described by [10] as ‘making the whole into something more than the parts alone imply’. However, similar to Phase 5, there has been no clear guidance on how to carry out this phase. During this phase, the studies are now viewed as a ‘whole’ with the aim of developing a framework [29, 31]. When writing about how the studies are related, reviewers can present this in a narrative and/or diagrammatic form [30]. Phase 6 can be broken down into the following two stages; (a) reciprocal and refutational synthesis and (b) line of argument synthesis.

##### (A) Reciprocal and refutational synthesis

This stage of the synthesis involves deciding whether the studies are sufficiently similar in their focus to allow for a reciprocal translation synthesis. Alternatively, the studies may refute each other in which case a refutational synthesis is conducted. It is possible to conduct both types of synthesis to discuss similar accounts (reciprocal translation synthesis) and also explore any contradictions between the studies (refutational synthesis) [23]. Generally, reciprocal translation syntheses are conducted more frequently in reviews than refutational syntheses and guidance on how to conduct a refutational synthesis is currently limited [23]. Below we first discuss how to carry out a reciprocal translation and then describe the way a refutational synthesis can be conducted. Referring to the translations table of data developed in the stages above allows reviewers to establish the relationship between the studies and consider how to approach a reciprocal and refutational synthesis.

*Reciprocal translation*

It is during this phase where the shared themes across the studies are summarised by juxtaposing the first and second order constructs. This leads to the generation of new concepts which provide a fuller account of the given phenomenon and resolve any contradictions [63]. These are known as the original third order constructs developed by the review authors and provide a new understanding of the phenomena [15]. To put briefly, this can be achieved by reading the primary data synthesis (Fig. 4) alongside the translations table (Fig. 5) and drawing out the main points to form the reciprocal translations and therefore developing the third order constructs. It is important to constantly check the summary and third order constructs you are developing against the translations table to ensure it is consistent with the original data.

*Refutational synthesis*

There are limited published examples of refutational synthesis [25, 45] as reviewers often focus on reciprocal translations [25]. Also reviewers may conduct a refutational synthesis, but not label it as such [23]. There are two published examples of refutational synthesis [43, 64]. This is not surprising given the lack of guidance available on how to conduct a refutational synthesis. The purpose of a refutational synthesis is to explore and explain the differences, exceptions, incongruities and inconsistencies in concepts across the studies [1, 24]. Refutational synthesis focuses on identifying, understanding and reconciling the contradictions, rather than developing concepts around the similarities. Similar to reciprocal translation, reviewers are required to refer back to the primary data synthesis and translations table in order to develop third order constructs. The contradictions between the concepts across the studies may be explained by differences in participants, settings or study design. During this phase, it is helpful to refer back to the study characteristics table as this can help provide context for interpretations and explanations [57]. It has been suggested that a refutational translation can be approached by placing two refutational concepts at either end of a continuum and proceed by analysing the differences between the concepts [22, 28]. In order to express the refutational findings, a narrative can be created so that the findings ‘are placed into context’ [28].

##### (B) A lines of argument synthesis

A lines of argument synthesis can then be created from the third order constructs, which involves ‘making a whole into something more than the parts alone imply’ (known as higher order interpretations) [10]. A lines of argument synthesis means that there is an ‘interpretation of the relationship between the themes, which further emphasises a key concept that may be hidden within individual studies in order to discover the whole from a set of parts’ [10]. This is classed as a further higher level of interpretative synthesis, and provides scope for developing new insights.

**Fig. 6:**
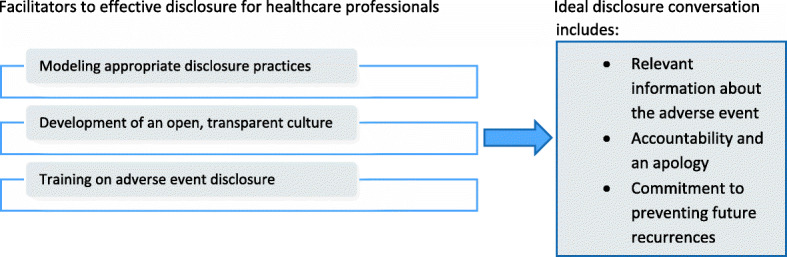
Example of a line of argument synthesis developed

A lines of argument synthesis is achieved by constant comparison of the concepts and developing a ‘grounded theory that puts the similarities and differences between the studies into interpretative order’ [10]. Practically, reviewers can approach this phase by reading through the reciprocal translations and noting down the similarities and differences between each of the third-order constructs. These notes can then be used to construct interpretations of how each third order construct relates to the others in the analysis. These relationships can then be represented using a diagram to aid understanding. Each of the reviewers can carry out this stage independently, and merge their findings as a team to produce the final line of argument synthesis. Diagrams can be used to develop the line of argument synthesis and it is suggested that discussions between team members are vital to this process [29, 30]. A lines of argument synthesis can be a useful way to bring together and explain the perspectives of two or more different groups and interpreting the relationship between the themes. This is particularly relevant for research in healthcare, where often the views of one or more groups are examined on a phenomenon (e.g. patients and healthcare professionals). An example of a line of argument synthesis from the worked example is presented in Fig. 6.

*Example*

We conducted separate reciprocal translations for the first- and second-order constructs relating to patients and healthcare professionals, resulting in third order constructs which related to solely either patients or professionals. Therefore, the synthesis process for our review consisted of three steps- (1) reciprocal translations of the patient studies to understand patients’ views and experiences of disclosure, (2) reciprocal translations of the healthcare professional studies to explore healthcare professionals views and experiences on disclosure and (3) a line of argument synthesis which contributed to the identification of both the key elements of an ideal disclosure desired by patients and the facilitators for healthcare professionals which can increase the likelihood of this taking place. We initially considered a refutational translation instead of a line-of-argument synthesis, but it was apparent during the synthesis that the concepts from the patient and healthcare professional studies were not contradictory in nature; rather they described alternate perspectives of the same phenomenon. Therefore we believed a line of argument synthesis was the most appropriate for the aim of our synthesis. During this stage of the analysis, we found it helpful to place all the third order constructs in a table to enable visual comparison (see Table 2).

**Table 2:** Examples of third order constructs

Third order constructs: Patient studies *(views on disclosure process)*	Third order constructs: Healthcare professional studies *(views on disclosure process)*	Third order constructs: Healthcare professional studies *(barriers to disclosure)*
Need for information	Sometimes economical with the truth	Difficulty of disclosure in a blame culture
Importance of sincere regret	Owning up without saying ‘I’m sorry’	Avoidance of litigation
Promise of improvement	To tell or to not tell? *-When anxiety may cause unnecessary anxiety* *-Outcome determines disclosure*	Disclosure is a learned skill
		Inconsistent guidance

The third order constructs should be theoretically rich. In our synthesis, although we found that the data we were dealing with was descriptive, it was rich descriptive data. This therefore provided us with sufficient detail to further interpret this and develop third order constructs [23]. The third order constructs we developed reflected the data we were dealing with, but allowed us to produce higher levels of analysis. Reviewers should take caution when dealing descriptive data. They need determine whether it is ‘thin descriptive data’ which could be problematic to further interpret due to lack of depth, or ‘rich descriptive data’ which can provide sufficient detail to be further interpreted [23].

#### Phase 7: expressing the synthesis

Reviewers should follow the eMERGE reporting guidance when writing up the synthesis [22] and the PRISMA guidelines may be used alongside this if systematic searches are conducted as many journals may require a PRISMA diagram [65]. In addition to these standard reporting methods as described by the eMERGE guidance [22] the final phase can be broken down into the following three stages; (a) summary of findings, (b) strengths, limitations & reflexivity and (c) recommendations and conclusions (refer to [22] where this phase is described in further detail).

## Discussion

Meta-ethnography is an evolving approach to synthesising qualitative research and is being increasingly used in healthcare research [29]. A meta-ethnographic approach offers a greater description of methods and higher-order interpretation (an overarching explanation of a phenomenon that goes beyond what the study parts alone imply), compared to a conventional narrative literature review [12]. The use of this approach can assist in generating evidence for healthcare staff, researchers and policy-makers. Although this approach is being used by numerous reviewers, transparency on how each of the stages should be conducted is still poor and there is a lack of clarity surrounding the exact stages reviewers utilise to reach their final synthesis [23]. The ultimate aim of qualitative research synthesis in healthcare is to contribute towards improvements in patient care and experience, as well as improving the processes for healthcare professionals involved [39]. In order for a meta-ethnography syntheses to be considered to be of high quality and useful, the meta-ethnographic approach needs to be rigorous and consistent. Therefore, a clear understanding of the steps included in a meta-ethnography is vital to produce a synthesis which is rigorous and comprehensive. Poorly reported methods of meta-ethnography can also make it challenging, particularly for early career qualitative researchers to conduct this synthesis. Therefore, we have provided a practical step-by-step guide to assist reviewers with conducting a meta-ethnographic synthesis of qualitative research. High quality qualitative research synthesis should not end with the final write up and further research needs to focus on how the impact of qualitative research can be maximised to improve healthcare.

Like any other method, the meta-ethnographic approach is not without its limitations. Within a meta-ethnography, although reviewers provide a synthesis, this is only one interpretation and as qualitative synthesis is subjective, several alternative interpretations are likely to be possible [66]. The subjective nature of a meta-ethnography may also affect the representativeness of the synthesis findings. To develop this guide, we searched for articles in a number of ways which is described in detail in the methods section. However, as a systematic literature search was not conducted to identify articles for the development of this guide, there is the potential that this may have resulted in the exclusion of some articles. Whilst we have provided guidance on how to conduct a meta-ethnographic synthesis, it is important to note that this is a flexible guide, which researchers can utilise and adapt the stages, according to their own research questions and the phenomenon under study. Some of the steps and challenges described in this guide hold true for systematic reviews in general. However, this guide aimed to offer practical step-by-step guidance on how to conduct meta-ethnography for even those researchers who may not be experienced in conducting systematic reviews as well as being unfamiliar with a meta-ethnographic approach. This guide was developed to assist with conducting a meta-ethnography within healthcare research. Although this guide would be potentially useful beyond healthcare research, there might be additional challenges and considerations in other research fields which may not be fully captured in this guide.

## Conclusions

There was previously a lack of step-by-step guide to meta-ethnography conduct. In this paper, we have filled this gap by providing a practical step-by-step guide for conducting meta-ethnography based on the original seven steps as developed by Noblit & Hare [10]. We have incorporated adaptations and developments by recent publications and we provide detailed annotations, particularly for stages 4–6 which are often described as being the most challenging to conduct, yet the least amount of guidance is provided for conducting these stages. We have described each stage in relation to one of the previous meta-ethnographies we have conducted to aid understanding, and allows the reader to follow on from one step to the next easily.

## Data Availability

The datasets used and/or analysed during the current study are available from the corresponding author on reasonable request.
